# Supplementary material for: Genome-Wide Identification and Analysis of the Growth-Regulating Factor Family in Zanthoxylum armatum DC and Functional Analysis of ZaGRF6 in Leaf Size and Longevity Regulation
Source: Int J Mol Sci. 2022 Aug 12;23(16):9043. doi: 10.3390/ijms23169043 (PMC9409285; doi:10.3390/ijms23169043)
Supplement: Supplementary file 1 [file ijms-23-09043-s001.zip › ijms-1842838-supplementary.pdf]

International Journal of
Molecular Sciences

MDPI

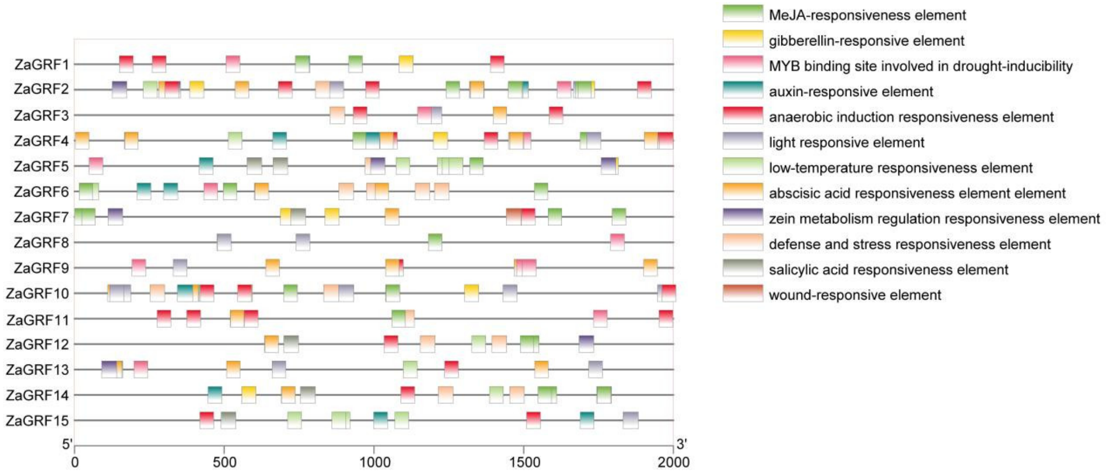

Figure S1. Cis-acting elements in the promoters of the *GRF* gene family.

Table S1. Primers for the qRT-PCR investigation of *ZaGRF* genes.

**Genes**	**Primer sequence（5' to 3'）**		**Amplicon size (bp)**
	**Forward primer**	**Reverse primer**	
ZaActin	GTGAGCCACACAG-TACCCAT	GGTGAAAGAGTACCCACGCT	129
ZaGRF1	ACTCAGTTGGTAC-GCGTGAG	TGTAACACGCCGTGCACTAT	221
ZaGRF3	CTCACACTCGAAGTG-CAGGT	GGCAA-GCCAAATGGATGCAA	208
ZaGRF4	GAGACACCAGCAG-CATGAGT	AGATGACCCAGATGAGGGCT	115
ZaGRF5	GGAAGGTGCAGTTGAC-GAGA	AGCACTTTCCCAGCCACAAT	150
ZaGRF6	GGAAGGAGGGCATCAC-CATT	TGCATGTGGCGTTCACAGTA	296
ZaGRF7	AA-GCTTTCAGAACCCGCCTT	AGTTGTTGTTACGCCCCTGT	259
ZaGRF8	TGCATTCATTTGGCTTGCCC	CGGCCCCGATTTATATGCCT	196
ZaGRF15	ACAAGCCCAC-CGAATCAACA	TGCTGGTGTTTTGGAGCCAT	209
NbActin	ACTGAA-GCGCCTCTTAACCC	AGACGGAGAATGG-CATGTGG	218
NbARR4	GTCCGACGAG-TCATCAACCC	GAGATGAAGTGGATGGCGGT	122
NbCRF2	AACGGAAGTGTTAG-TGGCGT	GTACACGTCGCAGTGGATCT	208
NbCKX1	AGCAGCAA-GAGGTCATGGTC	CGAGGGTAGCACGAA-GAACA	206

NbGLK1	AGCTTCGGATCTT-GGTTCGG	GCAATTCTGGCGTCCAATCC	203
NbPORB	CTGGAAACGTAC-CGCCAAAG	CGGGATAGAGGGAC-GCAAAG	213

Table S2. Subcellular localization and secondary structure prediction of ZaGRF proteins.

**Gene**	**Subcellular localization**	**Alpha helix**	**Exteneded strand**	**Random coil**
ZaGRF1	Nucleus	17.18	10.42	69.01
ZaGRF2	Extracellular matrix	12.01	19.88	59.45
ZaGRF3	Nucleus	15.46	12.65	67.47
ZaGRF4	Nucleus	19.79	9.38	69.01
ZaGRF5	Nucleus	19.72	15.85	58.10
ZaGRF6	Nucleus	12.61	10.81	72.37
ZaGRF7	Nucleus	22.18	11.60	61.43
ZaGRF8	Nucleus	18.87	9.20	69.02
ZaGRF9	Nucleus	18.75	10.68	68.75
ZaGRF10	Nucleus	19.16	12.07	66.93
ZaGRF11	Nucleus	19.18	8.95	70.08
ZaGRF12	Extracellular matrix	16.46	11.39	70.04
ZaGRF13	Nucleus	12.13	12.13	72.06
ZaGRF14	Extracellular matrix	20.08	10.74	66.20
ZaGRF15	Extracellular matrix	21.41	14.98	58.72

Table S3. The members of GRFs in *Z. armatum*, *C.sinensis*, *A.thaliana* and *O.sativa.*

Genes	Gene ID	Genes	Gene ID	Genes	Gene ID
ZaGRF1	Zardc16696.t1	*AtGRF1*	AT2G22840	*CsGRF7*	Cs_ont_7g017420
ZaGRF2	Zardc21503.t1	*AtGRF2*	AT4G37740	*OsGRF1*	LOC_Os02g53690
ZaGRF3	Zardc26531.t1	*AtGRF3*	AT2G36400	*OsGRF2*	LOC_Os06g10310
ZaGRF4	Zardc29111.t1	*AtGRF4*	AT3G52910	*OsGRF3*	LOC_Os04g51190
ZaGRF5	Zardc30654.t1	*AtGRF5*	AT3G13960	*OsGRF4*	LOC_Os02g47280
ZaGRF6	Zardc37188.t1	*AtGRF6*	AT2G06200	*OsGRF5*	LOC_Os06g02560
ZaGRF7	Zardc37898.t1	*AtGRF7*	AT5G53660	*OsGRF6*	LOC_Os03g51970
ZaGRF8	Zardc42395.t1	*AtGRF8*	AT4G24150	*OsGRF7*	LOC_Os12g29980
ZaGRF9	Zardc45509.t1	*AtGRF9*	AT2G45480	*OsGRF8*	LOC_Os12g29980
ZaGRF10	Zardc49103.t1	*CsGRF1*	Cs_ont_1g019360	*OsGRF9*	LOC_Os03g47140
ZaGRF11	Zardc50859.t1	*CsGRF2*	Cs_ont_1g023250	*OsGRF10*	LOC_Os02g45570
ZaGRF12	Zardc52135.t1	*CsGRF3*	Cs_ont_3g022280	*OsGRF11*	LOC_Os07g28430
ZaGRF13	Zardc52753.t1	*CsGRF4*	Cs_ont_5g030480	*OsGRF12*	LOC_Os04g48510
ZaGRF14	Zardc53033.t1	*CsGRF5*	Cs_ont_5g050440		
ZaGRF15	Zardc54515.t1	*CsGRF6*	Cs_ont_6g007580		

Table S4. The gene pairs among *Z. armatum, A. thaliana, C. sinensis* and *O. sativa.*

Gene pairs		Species
ZaGRF3	*ZaGRF8*	Za-Za
ZaGRF7	Zardc38469.t1	Za-Za
ZaGRF2	*AtGRF9*	Za-At
ZaGRF3	*AtGRF1*	Za-At
ZaGRF8	*AtGRF1*	Za-At
ZaGRF5	*AtGRF6*	Za-At
ZaGRF5	*AtGRF5*	Za-At
ZaGRF3	*AtGRF2*	Za-At
ZaGRF8	*AtGRF2*	Za-At
ZaGRF5	*OsGRF*1	Za-Os
ZaGRF2	Cs_ont_5g002060	Za-Cs
ZaGRF2	Cs_ont_5g041030	Za-Cs
ZaGRF3	*CsGRF2*	Za-Cs
ZaGRF5	Cs_ont_1g019360	Za-Cs
ZaGRF8	*CsGRF2*	Za-Cs
